# Birthweight, Maternal Weight Trajectories and Global DNA Methylation of LINE-1 Repetitive Elements

**DOI:** 10.1371/journal.pone.0025254

**Published:** 2011-09-28

**Authors:** Karin B. Michels, Holly R. Harris, Ludovic Barault

**Affiliations:** 1 Obstetrics and Gynecology Epidemiology Center, Department of Obstetrics, Gynecology and Reproductive Biology, Brigham and Women's Hospital, Harvard Medical School, Boston, Massachusetts, United States of America; 2 Department of Epidemiology, Harvard School of Public Health, Boston, Massachusetts, United States of America; National Institute on Aging, United States of America

## Abstract

Low birthweight, premature birth, intrauterine growth retardation, and maternal malnutrition have been related to an increased risk of cardiovascular disease, type 2 diabetes mellitus, obesity, and neuropsychiatric disorders later in life. Conversely, high birthweight has been linked to future risk of cancer. Global DNA methylation estimated by the methylation of repetitive sequences in the genome is an indicator of susceptibility to chronic diseases. We used data and biospecimens from an epigenetic birth cohort to explore the association between trajectories of fetal and maternal weight and LINE-1 methylation in 319 mother-child dyads. Newborns with low or high birthweight had significantly lower LINE-1 methylation levels in their cord blood compared to normal weight infants after adjusting for gestational age, sex of the child, maternal age at delivery, and maternal smoking during pregnancy (p = 0.007 and p = 0.036, respectively), but the magnitude of the difference was small. Infants born prematurely also had lower LINE-1 methylation levels in cord blood compared to term infants, and this difference, though small, was statistically significant (p = 0.004). We did not find important associations between maternal prepregnancy BMI or gestational weight gain and global methylation of the cord blood or fetal placental tissue. In conclusion, we found significant differences in cord blood LINE-1 methylation among newborns with low and high birthweight as well as among prematurely born infants. Future studies may elucidate whether chromosomal instabilities or other functional consequences of these changes contribute to the increased risk of chronic diseases among individuals with these characteristics.

## Introduction

The Developmental Origins of Health and Disease (DOHaD) suggest lifelong health implications of fetal and maternal growth trajectories. Low birthweight, premature birth, maternal underweight and/or malnutrition, and intrauterine growth retardation (IUGR) have been related to an increased risk of hypertension, cardiovascular disease, type 2 diabetes mellitus, obesity and neuropsychiatric disorders later in life [1]–[6]. Conversely, high birthweight, maternal obesity, high weight gain during pregnancy, and gestational diabetes have been linked to future risk of cancer, obesity, and type 2 diabetes in the offspring [7], [8]. The mechanisms underlying these associations are poorly understood. Fetal “programming” may reset the growth hormone/insulin-like growth factor axis but longitudinal studies connecting early life hormonal parameters to adult disease outcomes are lacking. It has recently been suggested that epigenetic mechanisms may be important contributors to fetal programming [9], [10].

DNA methylation is an important component of the cells machinery to regulate gene expression and occurs primarily on cytosine residues in CpG dinucleotides [11]. About half of human genes contain CpG-rich regions termed CpG islands in their promoter regions. Most lone CpG dinucleotides are in the introns of repetitive elements [12]. While most CpG islands are unmethylated, permitting transcription, the lone CpGs in the repetitive elements are mostly methylated.

About 50% of the human genome is composed of repetitive sequences such as LINE (Long Interspersed Nuclear Elements) and SINEs (Short Interspersed Nuclear Elements), including Alu [13]. These largely non-coding regions have been highly conserved throughout evolution but have lost their ability to move or make copies of themselves. The retrotransposition process involves recombination and two DNA single-strand breaks in close proximity, increasing the risk for chromosomal breaks, translocations, recombinations, and deletions [14]. The evolutionary younger subfamilies such as LINE-1 can still transcribe when activated. LINE-1 methylation decreases with age and hypomethylation of LINE-1 has been linked to various cancer types, possibly by contributing to chromosomal instability, and may be an early marker or a prognostic indicator of disease [15], [16].

DNA methylation marks are established *in utero* and the resulting methylation pattern is largely preserved through subsequent cell divisions through maintenance methylation [17]. Initial epigenetic reprogramming occurs during gametogenesis, allowing primordial germ cells to differentiate into mature oocyte and sperm [18]. After fertilization, male pronucleus and zygote undergo another round of demethylation which restores totipotency, followed by a genome-wide *de novo* methylation which contributes to cell fate commitment of the first cell lineages in preimplantation development [17], [19]–[21]. These reprogramming sequences makes the intrauterine period a target for environmental and metabolic factors that may affect the establishment of cytosine methylation.

The epigenetic signatures of the cord blood and the placenta need to be interpreted in the context of their different embryonic origins. The placenta is composed of extraembryonic tissue and develops upon implantation of the blastocyst the maternal endometrium. The outer layer of the blastocyst becomes the trophoblast which forms the outer layer of the placenta. The inner blastomeres form the embryonic tissue. Disruption of the epigenetic profile in the gametes may affect inner and outer blastomeres to the same degree, and hence be reflected in both cord blood and placenta tissue. Disruption after fertilization may affect the outer blastomeres of the preimplantation embryo and be reflected only in the placental tissue. The placenta has metabolic and endocrine activity. It produces hormones which maintain the pregnancy, stimulate growth of the fetus, and increase transfer of these nutrients to the fetus. The perfusion of the intervillous spaces of the placenta with maternal blood allows the transfer of nutrients and oxygen from the mother to the fetus and the transfer of waste products and carbon dioxide back from the fetus to the mother. Altered regulation of these physiologic processes due to differences in gene expression may affect fetal growth.

Aberrant DNA methylation, including global hypomethylation of DNA from peripheral blood leukocytes, has been linked to various chronic diseases including cancer and cardiovascular disease [22], [23]. The distribution of global methylation and frequency of hypomethylation at birth has not been studied. Whether changes in DNA methylation occur prenatally and predispose to disease, hence provide a mechanistic explanation for the DOHaD observations remains to be determined. Since birthweight is the best studied perinatal non-genetic marker of adult disease susceptibility we explored the association between birthweight and its predictors such as maternal prepregnancy BMI and maternal weight gain during pregnancy and LINE-1 methylation. We used data and biospecimens from the Epigenetic Birth Cohort at Brigham and Women's Hospital, Harvard Medical School, in Boston to explore the association between trajectories of fetal and maternal weight and LINE-1 methylation in 319 mother-child dyads.

## Results

Cord blood samples were available from all 319 newborns included in this study and placenta samples were available from 316 of them. Maternal and infant characteristics of the study population are provided in Table 1. Median LINE-1 methylation was 80% (range 75.8–84.3%) in cord blood and 51% (range 41.3–70.0%) in placental tissue. The infant with the highest LINE-1 methylation in placental tissue of 70% was characterized by a low birthweight of 2300 grams; when excluding this outlier the range of LINE-1 methylation in placenta was 41.3–59.9%.

**Table 1:** Characteristics of the 319 mother-infant dyads of the Epigenetic Birth Cohort included in this study.

Characteristic	Median/Percent	Range (minimum – maximum)
Percent LINE-1 methylation		
Cord blood (n = 319)	80	75.8–84.3
Placenta (n = 316)	51	41.3–70.0
Maternal characteristics		
Age at delivery (yrs)	32	18–45
Smoking (prior to or during pregnancy) [%]	6.9/7.0*	
Gestational diabetes [%]	9.7/9.8*	
Race/ethnicity [%]		
Non-Hispanic White	63.0/63.0*	
Hispanic	15.1/15.2*	
Asian/Pacific Islander	8.5/8.5*	
Black	12.2/12.0*	
Other	1.3/1.3*	
Pre-pregnancy BMI (kg/m^2^)	23.5*	17.5–40.8
Weight gain (lbs)	33	2–82
Infant characteristics		
Female [%]	48.0/48.1*	
Preterm (<37 weeks) [%]	11.9/11.7*	
Birthweight (g)	3433.5/3430*	1460–5395
Placenta weight/birthweight ratio	0.2	0.1–0.8

* First value: percent among study population with cord blood samples, second value: among study population with placenta samples.

Newborns with low or high birthweight had significantly lower LINE-1 methylation levels of their cord blood DNA compared to normal weight infants after adjusting for gestational age, maternal age at delivery, maternal smoking prior to or during pregnancy, ethnicity of the mother, and sex of the child, but the magnitude of the difference was small (Table 2). Infants born prematurely also had lower LINE-1 methylation levels of cord blood DNA compared to term infant, and this difference, though small, was statistically significant (p = 0.004). LINE-1 methylation level in the cord blood DNA decreased by about 3 percent per unit increase in the birthweight/placenta ratio (p = 0.058). Neither maternal prepregnancy BMI nor gestational weight change was significantly associated with LINE-1 methylation in the cord blood (Table 2). Gestational diabetes and maternal smoking prior to or during pregnancy, both of which affect fetal growth, were not associated with LINE-1 methylation (p = 0.63 and p = 0.58, respectively). Ethnicity of the mother (p for interaction = 0.31) or sex of the infant (p for interaction = 0.37) did not modify the associations observed.

**Table 2:** Maternal and fetal weight trajectories and LINE-1 global methylation among the 319 mother-infant dyads of the Epigenetic Birth Cohort included in this study.

Weight Trajectory		Cord Blood			Placenta		
	Number of Dyads	Difference in % Methylation^a^	95% CI for Difference in Methylation^a^	p-value^a^	Difference in % Methylation^a^	95% CI for Difference in Methylation^a^	p-value*
Birthweight (g)^b^							
<2500	29	−0.82	−1.42 to −0.23	0.007	1.41	0.18 to 2.63	0.025
2500–3999	227	0 (Ref)			0 (Ref)		
4000+	62	−0.43	−0.84 to −0.03	0.036	−0.14	−0.97 to 0.69	0.74
Preterm birth							
No	281	0 (Ref)			0 (Ref)		
Yes	38	−0.73	−1.22 to −0.24	0.004	−0.03	−1.03 to 0.98	0.96
Birthweight/placenta weight ratio (per increase in 1 unit)	319	−2.88	−5.86 to −0.10	0.058	−3.14	−9.14 to 2.87	0.30
Prepregnancy BMI (kg/m^2^)							
<20	43	0.09	−0.35 to 0.53	0.68	−0.25	−1.14 to 0.64	0.58
20–29.9	221	0 (Ref)			0 (Ref)		
30+	52	0.42	0.01 to 0.84	0.05	0.38	−0.47 to 1.22	0.38
Gestational weight change^c^							
<Recommended^d^	46	0.03	−0.48 to 0.54	0.90	0.07	−0.95 to 1.09	0.89
Recommended^d^	99	0 (Ref)			0 (Ref)		
>Recommended^d^	171	−0.03	−0.39 to 0.34	0.88	0.11	−0.63 to 0.85	0.77

a Linear regression models were adjusted for maternal age at delivery, maternal ethnicity, maternal smoking prior to or during pregnancy, and for the sex of the child.

b Additionally adjusted for preterm birth.

c Additionally adjusted for prepregnancy BMI.

d Based on the Institute of Medicine 2009 guidelines for gestational weight gain.

Weight trajectories were less correlated with LINE-1 methylation in fetal placental tissue (Table 2). The only statistically significant association emerged for low birthweight which was positively related to LINE-1 methylation in the placenta relative to normal birthweight (p = 0.025).

When we repeated the analyses among term infants the results did not appreciably change. However, the association between low birthweight and LINE-1 methylation in placental tissue became stronger with a difference of 2.6% methylation (p = 0.0014) compared to infants with normal birthweight.

## Discussion

In this largest study to date on birthweight and global methylation we found low and high birthweight and preterm birth to be significantly associated with a reduced LINE-1 methylation level in cord blood. We did not find important associations between maternal weight trajectories and global methylation of the cord blood or fetal placental tissue.

To our knowledge only two prior studies have considered the association between fetal growth trajectories and LINE-1 methylation. Bourque and colleagues found no difference in LINE-1 methylation in placenta samples from 13 IUGR pregnancies (50.0%) compared with 22 normal pregnancies (49.6%) [24]. In 12 fetal cord blood samples, Fryer, *et al.* found higher LINE-1 methylation levels among samples from higher birthweight infants but comparison groups were generated indirectly by hierarchical clustering [25].

Other studies on fetal growth trajectories and DNA methylation have employed a genome-wide approach or focused on imprinted genes, but all were afflicted by limited samples sizes. Using a microarray approach, Einstein and colleagues compared cord blood samples from five IUGR and five normal pregnancies and identified methylation differences at a limited number of loci [26]. Other authors identified differences in methylation and expression of selected imprinted genes in placenta and cord blood of IUGR or low birthweight compared to normal weight infants [27]–[30]. High birthweight has also been associated with increased promoter methylation of the glucocorticoid receptor gene in human placenta [31].

A decrease in LINE-1 methylation may contribute to the retro-transposition of the functional forms of this repetitive element. This may lead to mutagenesis by insertion, with consequences for gene regulation or even alteration of gene coding sequences. Hypomethylation of non- functional forms of this repetitive element may also affect chromatin condensation and increase the risk for chromosomal breaks, chromosomal instability, and chronically aberrant immune responses [32], [33]. These mechanisms may underlie the observed associations between global hypomethylation and cancer [22], [34].

The fetus is equipped with developmental plasticity, allowing adjustment to a range of intrauterine conditions. Adverse conditions beyond this range such as intrauterine starvation may result in reprogramming: stable alterations in epigenetic or other states that optimize chances for survival in the short term but may create suboptimal conditions for long-term health decades later. We found that low and high birthweight and premature birth were associated with reduced methylation in repetitive elements. Whether the modest changes observed have any functional relevance remains unclear but they may be a piece in the mechanistic puzzle of the well established association of these fetal growth markers with long-term health. Even small differences in LINE-1 methylation have been associated with the prevalence of disease [35].

No study has directly explored epigenetic changes as causal link between birthweight as a marker of intrauterine events and chronic disease outcomes in adulthood in humans. This void is likely due to the logistic difficulties in obtaining relevant blood or tissue samples at birth and at disease diagnosis from the same person which would be necessary to connect the perinatal marker and adult disease outcome to demonstrate a permanent perinatally induced epigenetic alteration. Epigenetic alterations in adulthood were found among individuals periconcentionally exposed to the Dutch Famine but whether these modifications were present at birth and whether they alter disease risk has not been examined [36].

The temporal relation between fetal growth and epigenetic variation at birth remains unclear. Birthweight and placental weight are influenced by factors operating throughout pregnancy [37]. While the DNA methylation pattern is initially set at the blastocyst state [21], it is possible that the intrauterine environment thereafter impacts upon the methylation profile. Birthweight and placental weight may be markers of the intrauterine environment that could affect the epigenetic profile present at birth. In the context of DOHaD, adverse intrauterine conditions may lead to permanent changes in the epigenome. We considered global methylation the dependent variable in our regression analyses which allowed us to simultaneously adjust for potential confounding variables.

The observed median LINE-1 methylation in placental tissue of 51% in our population is consistent with previous studies [24]. Cytosine methylation levels in normal tissues have been reported to be lowest in the placenta [38]. The low LINE-1 methylation in placental tissue and the observed fairly large interindividual variation in our study population may reflect greater plasticity of this organ in accommodating a variety of intrauterine conditions than blood and other tissues. Heterogeneity in cell types in placental tissue may also contribute to interindividual variation. Conversely, placental samples obtained at the end of this organ's life cycle, may reflect its decreasing activity. We observed higher LINE-1 methylation in the placenta of low birthweight infants. This may reflect the task of the placenta to maximize nutrition to the fetus. For a less well-nourished fetus the placenta may maintain a higher metabolic rate until birth.

Our study is the largest to date on the association between birthweight and repetitive element methylation. It is also the first to consider the association with placental weight and with maternal pregnancy weight trajectories. Given the global increase in pre-pregnancy body mass index, any effects on the infant would be of interest.

In conclusion, we found significant differences in cord blood global methylation among newborns with low and high birthweight as well as among prematurely born infants. Future studies may elucidate whether instabilities or other functional consequences of these changes contribute to the increased risk of chronic diseases among individuals with these characteristics.

## Materials and Methods

### Ethics Statement

The study protocol was approved by the Institutional Review Board of the Brigham and Women's Hospital, Boston, Massachusetts.

### Study Population

The Epigenetic Birth Cohort comprises 1941 mother-child dyads. Data and biospecimens were collected between June 2007 and June 2009 on the labor and delivery floor of the Department of Obstetrics, Gynecology and Reproductive Biology at Brigham and Women's Hospital in Boston. Pregnant women were requested to complete a 2-page questionnaire and asked for permission to abstract information from their pregnancy charts and to collect samples from umbilical cord and placenta after detachment for research purposes. From this cohort we sampled 319 mother-infant dyads from the base population oversampling pairs with low (<20 kg/m^2^) and high (30+ kg/m^2^) pre-pregnancy body mass index (BMI), low (< = 20 lbs) and high (50+ lbs) gestational weight gain, and low (<2500 g) and high (4000+ g) birthweight. Participants were frequency matched on maternal age (+/−2 years) and folic acid supplement use (pre- and post-conception intake/post-conception intake only).

### Maternal and Fetal Characteristics

Data on maternal date of birth, height, prepregnancy weight, gestational weight gain, gestational age, birthweight, and sex of the newborn were abstracted from the pregnancy charts. Prior to delivery, women were asked to complete a questionnaire and provide information about their race and ethnicity, height, weight prior to pregnancy, weight gain at each trimester and in total at the end of pregnancy, vitamin supplementation prior to conception and during pregnancy, and smoking habits and alcohol consumption. If medical record information was missing for height, prepregnancy weight, or gestational weight gain, questionnaire data were supplemented. Placental weight was directly measured.

### Biospecimen Collection

After umbilical cord and placenta were detached from mother and child, cord blood was collected from the base of the cord and divided into a PaxGene RNA tube (Qiagen, Valencia, CA) and an EDTA tube. Placental weight was assessed at this time. Blood in EDTA tubes was processed immediately and the buffy coat and red blood cell fractions were stored at −80°C until further processing. Plasma has been stored in liquid nitrogen. Blood in the PaxGene tube was stored at −20°C until further processing. Placental tissue samples were harvested from the upper side of the placenta near the umbilical cord (consisting of predominantly fetal cells), near the cord from the lower layer, at the placenta perimeter from the upper layer, and at the placenta perimeter from the lower layer, for DNA and RNA isolation, respectively. Collected placenta tissues for DNA extraction were snap-frozen and stored in liquid nitrogen. Tissues for RNA extraction were stored in RNAlater (Ambion, Carlsbad, CA) at −20°C until further processing. For the present study placental tissue from the upper side of the placenta near the umbilical cord was used.

### DNA Isolation

DNA was isolated from the buffy coat using the QIAamp DNA Blood Mini Kit by Qiagen. DNA was isolated from snap-frozen placenta tissues using the QIAamp DNA Mini Kit from Qiagen.

### Global Methylation Assay

Global methylation was assessed using a LINE-1 bisulfite pyrosequencing assay. A total of 200 ng of DNA was bisulfite converted using the EZ DNA Methylation-Gold Kit according to the manufacturer's alternative protocol 2 (Zymo Research, Orange, CA, USA). LINE-1 was amplified using the Qiagen HotstarTaq plus MasterMix and the Pyromark Q24 LINE-1 assay (Qiagen). Pyrosequencing was performed on a Pyromark Q24 (Qiagen). The LINE-1 assay of the Pyromark Q24 tests 3 CG dinucleotides, and verifies the completion of the bisulfite treatment by testing a cytosine outside a CG dinucleotide. The global methylation ratio was calculated using the mean of the three dinucleotides. All assays (starting with the bisulfite conversion) were done in duplicate. To optimize the precision of the experiments, the difference between the two replicates were compared to the standard deviation calculated from all samples of the same tissue. If the difference between the two replicates was larger than two standard deviations, bisulfite treatment, PCR, and pyrosequencing were performed a third time for the respective samples. The quantitative performance of the pyrosequencing assays was verified by including methylation standards comprised of known proportions of unmethylated and fully methylated DNA. Average reproducibility (absolute difference between the means of replicates) of bisulfite pyrosequencing for LINE-1 assays in our laboratory was within 3% variability.

### Statistical Analysis

Percent methylation was averaged across the 3 CpG sites (calculating the mean of the replicate means) for cord blood and placenta, respectively. Percent methylation was used as a continuous variable. Linear regression was used to model the association between LINE-1 methylation and maternal and fetal weight trajectories while simultaneously adjusting for maternal age at delivery (continuous) [39], maternal ethnicity [40], maternal smoking prior to or during pregnancy, and sex of the child [41]. Model assumptions were verified through residual plots, which were normally distributed. The weight trajectories were coded as follows: Birthweight: <2500, 2500–3999, 4000+g; premature birth (dichotomous); birthweight/placenta weight ratio (continuous); maternal pre-pregnancy BMI: <20, 20–29.9, 30+ kg/m^2^; gestational weight gain: less than recommended, recommended, more than recommended according to the Institute of Medicine Guidelines, which state that healthy American women at a normal BMI of 18.5 to 24.9 kg/m^2^ should gain 25 to 35 pounds during pregnancy, underweight women (BMI<18.5 kg/m^2^) should gain 28 to 40 pounds, overweight women (BMI of 25 to 29.9 kg/m^2^) should gain 15 to 25 pounds, and obese women (BMI>30) should limits weight gain to 11 to 20 pounds [42]. Possible effect modification by ethnicity of the mother or sex of the child was assessed using a Wald test.

All statistical significance tests were 2-sided and an alpha-level of 0.05 was used.
